# Esomeprazole for the treatment of erosive esophagitis in children: an international, multicenter, randomized, parallel-group, double-blind (for dose) study

**DOI:** 10.1186/1471-2431-10-41

**Published:** 2010-06-11

**Authors:** Vasundhara Tolia, Nader N Youssef, Mark A Gilger, Barry Traxler, Marta Illueca

**Affiliations:** 1 Wayne State University, Detroit, MI, USA; 2 Atlantic Health, Morristown, NJ, USA; 3 Baylor College of Medicine, Houston, TX, USA; 4 AstraZeneca LP, Wilmington, DE, USA; 5 Current Address: Providence Hospital, Southfield, MI, USA; 6 Current Address: AstraZeneca LP, Wilmington, DE, USA

## Abstract

**Background:**

Acid suppression with a proton pump inhibitor is standard treatment for gastroesophageal reflux disease and erosive esophagitis in adults and increasingly is becoming first-line therapy for children aged 1-17 years. We evaluated endoscopic healing of erosive esophagitis with esomeprazole in young children with gastroesophageal reflux disease and described esophageal histology.

**Methods:**

Children aged 1-11 years with endoscopically or histologically confirmed gastroesophageal reflux disease were randomized to esomeprazole 5 or 10 mg daily (< 20 kg) or 10 or 20 mg daily (≥ 20 kg) for 8 weeks. Patients with erosive esophagitis underwent an endoscopy after 8 weeks to assess healing of erosions.

**Results:**

Of 109 patients, 49% had erosive esophagitis and 51% had histologic evidence of reflux esophagitis without erosive esophagitis. Of the 45 patients who had erosive esophagitis and underwent follow-up endoscopy, 89% experienced erosion resolution. Dilation of intercellular space was reported in 24% of patients with histologic examination.

**Conclusions:**

Esomeprazole (0.2-1.0 mg/kg) effectively heals macroscopic and microscopic erosive esophagitis in this pediatric population with gastroesophageal reflux disease. Dilation of intercellular space may be an important histologic marker of erosive esophagitis in children.

**Trial Registration:**

D9614C00097; ClinicalTrials.gov identifier NCT00228527.

## Background

Gastroesophageal reflux disease (GERD) increasingly is recognized in young children. A recent retrospective population-based cohort study in Rochester, MN, found that the incidence of GERD in children aged < 5 years was 0.9/1,000 person-years [1]. Data on the prevalence and severity of erosive esophagitis (EE) in young children are limited. The prevalence of endoscopy- and biopsy-proven EE in one study was 29% in 209 patients with GERD aged 18 months to 10 years who had no neurologic abnormalities or congenital esophageal anomalies [2]. A retrospective review of the Pediatric Endoscopy Database System-Clinical Outcomes Research Initiative (PEDS-CORI) demonstrated that, of 7,188 children aged ≤ 18 years who underwent endoscopy, 12.4% had EE [3].

Although endoscopy is a valuable tool in the diagnosis of pediatric GERD and EE that provides macroscopic evidence of erosions, histology is important because abnormalities may be present without visible lesions on endoscopy. The North American Society for Pediatric Gastroenterology, Hepatology, and Nutrition (NASPGHAN) guidelines for pediatric GERD recommend esophageal biopsy in conjunction with diagnostic endoscopy [4]. If erosions are identified, histology is not considered mandatory for routine diagnosis of GERD. On histology, esophagitis is diagnosed by the presence of epithelial hyperplasia, intraepithelial inflammation, vascular dilatation in papillae, balloon cells, and ulceration [5]. Dilated intercellular spaces have been described as an additional morphologic feature of GERD and esophagitis in infancy and childhood [6]. Mucosal biopsies also are recommended but not mandatory in current pediatric endoscopy practice to exclude potentially confounding diagnoses, such as eosinophilic or infectious esophagitis and, less commonly, Barrett's esophagus [4].

Acid suppression with a proton pump inhibitor (PPI) is standard treatment for GERD and EE in adults [7] and increasingly is becoming first-line therapy for children aged 1-17 years [4]. Currently three PPIs are approved by the US Food and Drug Administration for the treatment of EE in children: esomeprazole (1-17 years), omeprazole (2-16 years), and lansoprazole (1-17 years). Findings from direct comparative studies in adults show that esomeprazole more effectively heals EE in adults than omeprazole [8,9], lansoprazole [10,11], and pantoprazole [12]; however, similar studies have not been conducted in pediatric populations. In this report, we describe the healing of EE after esomeprazole treatment in children aged 1-11 years. Although not a planned objective, this study allowed assessment of the usefulness of an adult classification system for EE in the pediatric population, the Los Angeles (LA) Classification System. In addition, because the literature lacks reports of histologic data from young children with GERD, baseline histology findings are reported here. The pharmacokinetic profile of esomeprazole in children aged 1-11 years has been published previously [13]. The primary safety and clinical outcomes of esomeprazole treatment of GERD from this study have been reported previously [14].

## Methods

### Study design and patients

The study design, methodology, eligibility criteria, patient characteristics, and safety assessments have been described in detail previously [14]. Children aged 1-11 years with endoscopically confirmed GERD (determined by endoscopy with or without biopsies) were screened and eligible to be enrolled in an international, multicenter, randomized, parallel-group, double-blind (for dose) study evaluating once-daily esomeprazole during 8 weeks of treatment. Patients with allergic or eosinophilic esophagitis, gastric ulcers, bleeding lesions, strictures, and Barrett's esophagus were excluded from the study. Erosive or histologic GERD was documented; however, EE was not required. Endoscopic findings were classified using the LA Classification System for EE (Table 1) [15]. As described in the literature [14], macroscopic evidence for GERD seen on endoscopy was documented and included hyperemia, ulcers, and nodularity. Patients with no visible or definitive lesions underwent a mucosal biopsy during baseline endoscopy for histologic confirmation of reflux esophagitis. Valid pediatric indicators of histologic reflux esophagitis were recorded, including the presence of intraepithelial eosinophils or neutrophils and increased basal cell layer thickness and papillary height [16,17]. Criteria for establishing and documenting a clinical diagnosis of GERD were consistent with the NASPGHAN guidelines [4].

**Table 1:** Los Angeles Classification System for erosive esophagitis [15]

LA grade	Description
A	≥1 Mucosal break ≤5 mm that does not extend between the tops of two mucosal folds
B	≥1 Mucosal break ≥5 mm long that does not extend between the tops of two mucosal folds
C	≥1 Mucosal break that is continuous between the tops of two or more mucosal folds but that involves < 75% of the esophageal circumference
D	≥1 Mucosal break, which involves ≥75% of the esophageal circumference

LA: Los Angeles.

Patients were assigned randomly to esomeprazole (Nexium^®^; AstraZeneca LP, Wilmington, DE) 5 or 10 mg (children ≥ 8 kg and < 20 kg) or 10 or 20 mg (children ≥ 20 kg) once daily for 8 weeks. For children aged < 6 years or for those who had difficulty swallowing the capsules, capsule contents could be mixed with 1 tablespoon of applesauce. Age-appropriate liquid antacid medication, MAALOX^® ^(aluminum hydroxide 225 mg/magnesium hydroxide 200 mg per 5 mL; Novartis Consumer, Parsippany, NJ) or equivalent, was allowed as rescue medication. Parents or guardians were instructed to administer rescue medication according to product labeling or as prescribed by the physician. Rescue medication use was recorded; when applicable, use in excess of the prescribed amount was reviewed with the parent or guardian at each visit and documented.

Institutional Review Boards at each participating center approved the study protocol (AstraZeneca study code D9614C00097; ClinicalTrials.gov identifier NCT00228527), and each patient's parent or guardian provided written informed consent with assent from the patient, where applicable, before any study-specific procedure was performed. Study procedures were conducted in accordance with the ethical principles of the Declaration of Helsinki and its amendments and with the International Conference on Harmonization and Good Clinical Practice guidelines.

### Assessments

All procedures including upper endoscopic evaluation were indicated clinically and represented standard practice at the local institution. Accordingly, no patient underwent endoscopy solely for study enrollment or other research purposes. An upper gastrointestinal endoscopy was performed during screening at the discretion of the investigator. Patients who had a previous endoscopic diagnosis of EE within 2 weeks of screening and were candidates for PPI therapy were not required to have an additional endoscopy if a full endoscopic report with photographic documentation was available. Histologic evidence was required for patients who were newly diagnosed with GERD if the patient did not have EE to confirm the presence of esophagitis. Endoscopy with biopsy was used to document the extent of EE, determine the presence of *Helicobacter pylori*, and rule out certain exclusionary conditions. For patients who had EE at baseline, a repeat endoscopy was planned after 8 weeks of esomeprazole treatment to document healing. GERD-related symptoms were reported by the parents or guardians on behalf of the patients. These symptoms were derived from the NASPGHAN guidelines [4] and included heartburn (burning feeling rising from the stomach or lower part of the chest toward the neck), acid regurgitation (perception of unpleasant-tasting fluid backing up into the throat and/or mouth), epigastric pain (perception of discomfort located in the central upper portion of the abdomen), vomiting (gastric contents are forced up to and out of the mouth), difficulty swallowing (difficulty passing anything through the pharynx or esophagus), and feeding difficulties (food refusal, choking with food/drink, and/or poor weight gain).

The LA Classification System was used to grade EE (Table 1) [15]. Other pediatric endoscopic GERD descriptors of esophagitis as reported in the literature were accepted, and appropriate histologic confirmation was obtained when indicated [18,19]. Per routine standards of pediatric medical practice [20], mucosal biopsy specimens were obtained during baseline endoscopy for histologic confirmation of GERD-related esophagitis in patients without visible or definitive lesions [21]. If needed, biopsy specimens were recommended to be taken from the distal esophagus, approximately 0.5 cm above the Z-line (squamocolumnar junction) based on the investigator's assessment of landmarks, and from any area with an abnormal appearance. Biopsy was optional for patients with endoscopically visible lesions, and specimens were obtained for medical reasons only at the discretion of the investigator. Biopsy specimens were evaluated at each study site for histologic findings, including the number of intraepithelial eosinophils and neutrophils [4,22,23], lymphocytes per high power field, elongated length of papillae (> 50% to 75% of epithelial thickness) [4,21,22], increased thickness of the basal cell layer (> 20% to 25% of total epithelial thickness) [4,21,22], and increased total epithelial thickness. Histologic changes were recorded [24-26] but not required as a research procedure. Histologic changes were characterized based on the standards from each pathologist's individual institution; findings were recorded on the case report form (e.g., those listed previously in Methods).

Safety was assessed by adverse events (AEs) spontaneously reported by the parent or guardian, reported in response to an open question from the investigator, or revealed by observation or change from baseline conditions or values in medical histories, physical examinations, vital signs, and clinical laboratory evaluations.

### Statistical analysis

Outcome analyses of EE healing were conducted on the intention-to-treat (ITT) population dataset. The ITT population was defined as all patients who had baseline and one or more postbaseline measurements, one or more ingested doses of study medication, and completion of a posttreatment endoscopy. EE was considered healed if no signs of erosion were observed on final endoscopy. The percentage of patients with healed esophageal erosions and the 95% confidence interval for the total were calculated by AstraZeneca (B.T.). The International Conference on Harmonization guideline E1 recommends randomization of ≥ 100 patients for the safety database of any drug. Therefore, the study was designed to randomize ≥ 100 patients to ensure that ≥ 40 patients in each age group would complete the study.

## Results

### Patient characteristics

**Figure 1:**
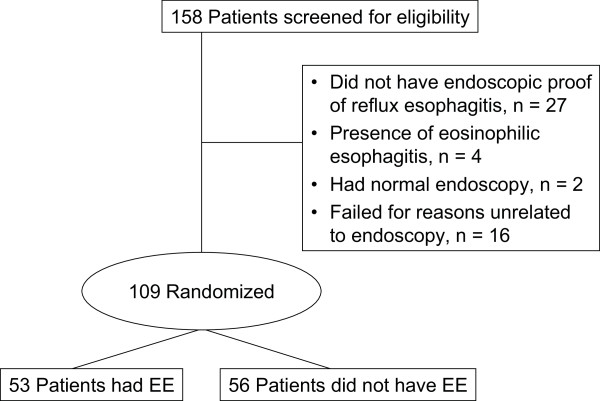
**Patient disposition**. EE: erosive esophagitis.

The study was conducted between October 2004 and November 2005 at 24 sites within Belgium (three sites), France (two sites), Italy (four sites), and the United States (15 sites). A total of 109 patients were randomized in the study and included in the ITT population. Of the 49 patients who failed the screening process, four had eosinophilic esophagitis, 27 had no endoscopic proof of reflux esophagitis, two had a normal endoscopy, and 16 were not related to endoscopy (Figure 1). Baseline demographic and nonphysical disease characteristics were similar across all treatment groups (Table 2). Fifty-two patients (47.7%) were aged 1-5 years, and 57 (52.3%) were aged 6-11 years. The mean age was 5.7 years. Height, weight, and body mass index also were similar across dose groups within each weight stratum (< 20 kg or ≥ 20 kg). Esomeprazole doses ranged 0.2-1.0 mg/kg. The most common presenting GERD symptoms at baseline were heartburn (52%), acid regurgitation (55%), and epigastric pain (55%) (Table 2). The distribution of patients met the study goal of ≥ 40 evaluable patients in each age group.

**Table 2:** Demographic and baseline disease characteristics of all patients enrolled (N = 109)

	Children < 20 kg		Children ≥ 20 kg	
		
Characteristic	Esomeprazole 5 mg (0.3-0.6 mg/kg) (n = 26)	Esomeprazole 10 mg (0.6-1.0 mg/kg) (n = 23)	Esomeprazole 10 mg (0.2-0.5 mg/kg) (n = 31)	Esomeprazole 20 mg (0.3-1.0 mg/kg) (n = 29)
Girls, n (%)	14 (53.8)	14 (60.9)	14 (45.2)	11 (37.9)

Mean age, years	2.1	2.5	8.5	8.3
Age in years, n (%)				
1	12 (46.2)	8 (34.8)		
2	6 (23.1)	5 (21.7)		
3	4 (15.4)	4 (17.4)		
4	2 (7.7)	3 (13.0)	1 (3.2)	2 (6.9)
5	1 (3.8)	2 (8.7)	1 (3.2)	1 (3.4)
6	1 (3.8)	1 (4.3)	2 (6.5)	0
7			5 (16.1)	3 (10.3)
8			5 (16.1)	9 (31.0)
9			8 (25.8)	6 (20.7)
10			3 (9.7)	6 (20.7)
11			6 (19.4)	2 (6.9)
Race, n (%)				
White	19 (73.1)	19 (82.6)	26 (83.9)	25 (86.2)
Black	7 (26.9)	4 (17.4)	5 (16.1)	3 (10.3)
Other	0	0	0	1 (3.4)
Mean height (range), cm	90.0 (70-109)	94.2 (80-119)	134.5 (108-168)	134.5 (112-159)
Mean weight (range), kg	12.8 (8-18)	14.1 (10-18)	35.5 (20-58)	34.5 (21-60)
Mean body mass index (SD), kg/m^2^	15.7 (2.1)	15.9 (1.7)	19.3 (4.8)	18.6 (3.9)
*Helicobacter pylori*-positive, n (%)	0	1 (4.3)	0	0
Symptoms at baseline, n (%)				
Heartburn	15 (57.7)	10 (43.5)	19 (61.3)	13 (44.8)
Acid regurgitation	18 (69.2)	11 (47.8)	20 (64.5)	11 (37.9)
Epigastric pain	17 (65.4)	13 (56.5)	15 (48.4)	15 (51.7)
Vomiting	13 (50.0)	7 (30.4)	3 (9.7)	5 (17.2)
Eating difficulties	15 (57.7)	13 (56.5)	9 (29.0)	7 (24.1)
Difficulty swallowing	6 (23.1)	8 (34.8)	5 (16.1)	6 (20.1)
Extraesophageal symptoms at baseline, n (%)	n = 12	n = 12	n = 16	n = 13
Hoarseness	4 (33.3)	4 (50.0)	4 (25.0)	7 (53.8)
Coughing	8 (66.7)	9 (75.0)	7 (43.8)	7 (53.8)
Gagging	5 (41.7)	6 (50.0)	2 (12.5)	4 (30.8)
Wheezing/stridor	1 (8.3)	1 (8.3)	2 (12.5)	0 (0)
Mean (range) esomeprazole dose, mg/kg	0.4 (0.3-0.6)	0.7 (0.6-1.0)	0.3 (0.2-0.5)	0.6 (0.3-1.0)

Of 109 patients randomized, 53 (49%) had EE at baseline and 56 (51%) had reflux esophagitis without EE (Table 3). Of 83 patients enrolled from study sites within the United States, 36 (43%) had EE; of 26 patients enrolled from European sites, 17 (65%) had EE. The proportion of patients with EE and other reflux esophagitis was distributed evenly across treatment groups. All but two patients had LA grades A or B EE (Table 3); one patient had grade C (< 20 kg/10-mg group), and the other had grade D (≥ 20 kg/20-mg group) [14]. Hiatal hernia was documented in seven children in the < 20-kg group and 12 children in the ≥ 20-kg group. Other esophageal abnormalities (e.g., hyperemia, esophageal ulcers, nodularity, prolapse gastropathy) were present in 55 children (n = 21, < 20 kg; n = 34, ≥ 20 kg) (Table 3). Of 109 patients in the ITT population, 107 had a biopsy. Investigators reported other gastric and duodenal abnormalities when detected. Other histologic findings were present in the esophagus at baseline (Table 4). Dilation of intercellular space was reported in 24% of patients.

**Table 3:** Endoscopic findings at baseline, n (%)

	Children < 20 kg		Children ≥20 kg		
			
Category	Esomeprazole 5 mg (0.3-0.6 mg/kg) (n = 26)	Esomeprazole 10 mg (0.6-1.0 mg/kg) (n = 23)	Esomeprazole 10 mg (0.2-0.5 mg/kg) (n = 31)	Esomeprazole 20 mg (0.3-1.0 mg/kg) (n = 29)	Total (N = 109)
Other reflux esophagitis	14 (54)	11 (48)	15 (48)	16 (55)	56 (52)

Erosive esophagitis	12 (46)	12 (52)	16 (52)	13 (45)	53 (49)
LA grade A	6 (23)	6 (26)	11 (36)	9 (31)	32 (29)
LA grade B	6 (23)	5 (22)	5 (16)	3 (10)	19 (17)
LA grade C	0	1 (4)	0	0	1 (1)
LA grade D	0	0	0	1 (3)	1 (1)
Hiatal hernia	4 (15)	3 (13)	8 (26)	4 (14)	19 (17)
Other abnormality*	11 (42)	10 (44)	16 (52)	18 (62)	55 (50)

*Abnormalities occurring in ≥4 patients were nodularity (n = 20 [18%]), erythema/hyperemia (n = 23 [21%]), edema (n = 11 [10%]), prominent esophageal folds (n = 11 [10%]), and friability (n = 4 [4%]).

LA: Los Angeles.

**Table 4:** Baseline histologic data of the esophagus, n (%)*

	Children < 20 kg		Children ≥20 kg		
			
Category	Esomeprazole 5 mg (0.3-0.6 mg/kg) (n = 26)	Esomeprazole 10 mg (0.6-1.0 mg/kg) (n = 23)	Esomeprazole 10 mg (0.2-0.5 mg/kg) (n = 31)	Esomeprazole 20 mg (0.3-1.0 mg/kg) (n = 29)	Total (N = 109)
Eosinophilic densification	4 (15)	3 (13)	8 (26)	12 (41)	27 (25)
Intraepithelial eosinophils^†^	5 (19)	9 (39)	13 (42)	13 (45)	40 (37)
Intraepithelial neutrophils^†^	5 (19)	1 (4)	6 (19)	3 (10)	15 (14)
Intraepithelial lymphocytes^†^	13 (50)	9 (39)	17 (55)	14 (48)	53 (49)
Elongated length of papillae	16 (62)	10 (44)	16 (52)	19 (66)	61 (56)
Increased thickness of basal cell layer	15 (58)	13 (56)	15 (48)	19 (66)	62 (57)
Increased total epithelial thickness	11 (42)	10 (44)	10 (32)	10 (34)	41 (38)
Dilation of intercellular spaces					
Absent/NR	21 (81)	21 (91)	24 (77)	17 (59)	83 (76)
< 25%	3 (12)	2 (9)	5 (16)	10 (34)	20 (18)
≥25%	2 (8)	0	2 (6)	2 (7)	6 (6)
Columnar epithelium					
Present	4 (15)	4 (17)	3 (10)	4 (14)	15 (14)
Not assessable	1 (4)	5 (22)	7 (23)	4 (14)	17 (16)
Cardia mucosa	2 (8)	3 (13)	1 (3)	4 (14)	10 (9)
Corpus mucosa	1 (4)	1 (4)	3 (10)	1 (3)	6 (6)
Diagnosis of microscopic reflux esophagitis	20 (77)	18 (78)	24 (77)	24 (83)	86 (79)

*107 Patients had a biopsy.

^†^Per high power field.

NR: not reported.

### Esophageal healing

**Figure 2:**
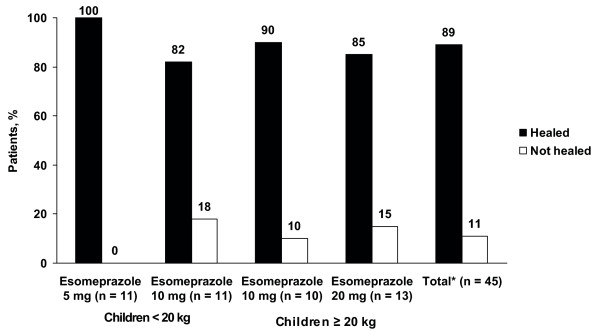
**Endoscopic healing status of erosive esophagitis after 8 weeks of esomeprazole treatment**. *Of 53 patients with erosive esophagitis at baseline, eight did not have a final endoscopy.

Of 53 patients with EE at baseline, eight did not undergo final endoscopies or were otherwise excluded from the ITT population because of premature study discontinuation (n = 4), revised diagnosis (n = 2), endoscopy performed outside the study timeframe (n = 1), or failure to give reason for not performing endoscopy (n = 1). Of 45 patients who had EE at baseline and underwent posttreatment endoscopy, EE was healed in 40 patients (89%) (Figure 2). Healing rates were similar across all esomeprazole dose groups. Healing of EE did not occur in five patients (11%): two patients in the 20-mg group were rated as improved but not completely healed (i.e., ≥ 1 LA grade improvement), and three patients in the 10-mg groups exhibited no change from baseline (n = 2, < 20 kg; n = 1, ≥ 20 kg). Patients whose EE was not healed received doses in the range of 0.17-0.66 mg/kg.

### Adverse events

Safety data were evaluable in 108 of 109 patients randomized and are described in detail elsewhere [14]. In brief, 13 AEs considered by the investigator to be related to esomeprazole treatment occurred in 10 of 108 patients (9.3%). The most commonly reported treatment-related AEs were diarrhea (2.8%; n = 3, 10-mg/< 20 kg group), headache (1.9%; n = 2, 10-mg/≥ 20 kg group), and somnolence (1.9%; n = 1, 5-mg/< 20 kg group and n = 1, 10-mg/< 20 kg group).

## Discussion

Based on this study, the use of esomeprazole across a wide dose range (0.2-1.0 mg/kg) daily for ≤ 8 weeks resulted in significant healing of macroscopic and histologic esophagitis in children aged 1-11 years. Few studies of PPI treatment of young children with GERD, including those with EE, are available [27-34]. The results of the present study provide additional evidence to support the safe use and tolerability of PPIs in children with GERD with or without EE [4].

Moreover, to our knowledge, this study is the first to prospectively report the use of the LA Classification System [15] to diagnose and document EE healing in young children. Several EE classification systems exist in the adult literature (e.g., Hetzel-Dent or Savary-Miller classifications), which have been adapted previously for pediatric studies [31,32]. Our results demonstrate that the LA Classification System can be used successfully to classify the severity of EE in children. The majority of children with EE in this study had LA grades A and B (29% and 17%, respectively). The LA classification system offered a simple, straightforward method to grade EE and document healing in the absence of erosions. The use of the LA classification system in children may allow for comparison between pediatric and adult data to unify our knowledge of healing of EE; however, it has not been validated yet for use in pediatric populations.

The doses used in this study were determined from the results of a pharmacokinetic study of esomeprazole in children aged 1-11 years, an extrapolation of the recommended adult esomeprazole doses using an exposure-response relationship reported previously [13], and the assumption that most children < 12 years weigh 8-60 kg. For ethical reasons in a population of children with confirmed GERD, this study did not include a placebo control group but was double blind to dosage. The lack of a control group is a potential limitation to this study. Furthermore, the assessment of the clinical outcome of EE was not controlled and was not the primary end point of the study; however, more importantly, the clinical benefit of esomeprazole in healing EE was documented. In addition, the small number of patients in each treatment group precluded a comparison between doses.

In the present study, the prevalence of EE (49%) in children aged 1-11 years was higher than that reported previously in children (12.4%) [3]. Baseline endoscopic and histologic data showed that 18% of patients had esophageal nodules, which have been shown to be a possible predictor of EE in the PEDS-CORI [18]. Our results suggest that dilation of intercellular space may be a potential histologic diagnostic criterion for EE [6]. Dilation of intercellular space was reported in 24% of the patients in this study (reporting this information was not mandatory), whereas 49% of patients had endoscopically confirmed EE. Although these potential histologic predictors of EE were identified in patients in this study, we cannot determine accurately the incidence of these potential markers because not every patient was evaluated for these histologic changes in a standardized manner. Determination of the true incidence of these markers in children is an area of future research. Large epidemiologic studies in children also are needed to determine the role of extraesophageal symptoms and concomitant conditions (e.g., asthma) in GERD.

The criteria for establishing and documenting endoscopically proven GERD for study entry were consistent with those previously recommended by NASPGHAN [4,20]. Endoscopic findings were required to be documented at study entry to allow for full characterization of the extent of GERD, including the presence and severity of EE and other gross findings. Furthermore, histologic assessment, when available, aided in excluding the diagnosis of other esophageal disorders, such as eosinophilic esophagitis. Although biopsy specimens were not evaluated by a central reader and therefore were not standardized, the histopathologic data obtained contribute to the existing sparse literature in this patient population. Additionally, the reason for a greater proportion of patients with EE in the European study sites compared with the United States study sites is not clear. The reasons for such geographic variation need to be studied further.

In this pediatric population, the clinical course and manifestations of the spectrum of GERD symptoms appear to be similar to those seen in adults. The current study continues to expand on the knowledge and potential management options that are available for young children with GERD and EE, and adult efficacy data may be extrapolated to this age group [3,4,35-37]. An 8-week treatment duration represents the approximate time needed for healing of EE in adults [8-12]. Guidelines for treatment of pediatric GERD recommend a 3-month course of acid suppression treatment for children who have GERD symptoms [4]. The results of the present study parallel the results from those of previous studies of PPIs in adults with EE and provide additional support for the use of esomeprazole treatment for EE in young children.

## Conclusion

The findings of this study showed that an 8-week course of esomeprazole treatment (0.2-1.0 mg/kg) healed esophageal erosions in 89% of children aged 1-11 years who had EE. Although the LA Classification System was used successfully to grade EE in this study, the development of a new pediatric-specific scoring system is suggested. For example, a complementary scoring system is needed to accommodate other pediatric endoscopic findings described in the literature, such as esophageal nodules [18]. Histologic assessment showed frequent mucosal damage in this population and provides further support for the use of histology to augment endoscopic findings in pediatric patients with GERD.

## Abbreviations

AE: adverse event; EE: erosive esophagitis; GERD: gastro esophageal reflux disease; ITT: intention-to-treat; LA: Los Angeles; NASPGHAN: North American Society for Pediatric Gastroenterology, Hepatology, and Nutrition; PEDS-CORI: Pediatric Endoscopy Database System-Clinical Outcomes Research Initiative; PPI: proton pump inhibitor.

## Competing interests

VT, MAG, and NNY have received grant/research support from AstraZeneca. MAG has served as a speaker and a consultant for TAP and AstraZeneca and has served as a speaker for Nestle. VT has received research grants from Wyeth, Johnson & Johnson, and GlaxoSmithKline and has served as a speaker for Takeda and SHS Nutritionals. NNY, BT, and MI are employees of AstraZeneca LP.

## Authors' contributions

VT made substantial contributions to conception and design, acquisition of data, and analysis and interpretation of data and was involved in drafting the manuscript and revising the manuscript for important intellectual content. NNY made substantial contributions to acquisition of data and analysis and interpretation of data and was involved in revising the manuscript for important intellectual content. MAG made substantial contributions to analysis and interpretation of the data and was involved in revising the manuscript for important intellectual content. BT made substantial contributions to conception and design and to analysis and interpretation of the data and was involved in drafting the manuscript and revising the manuscript for important intellectual content. MI made substantial contributions to conception and design, acquisition of data, and analysis and interpretation of data and was involved in revising the manuscript for important intellectual content. All authors read and approved the final manuscript.

## Pre-publication history

The pre-publication history for this paper can be accessed here:

http://www.biomedcentral.com/1471-2431/10/41/prepub
